# Indications of Peripheral Pain, Dermal Hypersensitivity, and Neurogenic Inflammation in Patients with Lipedema

**DOI:** 10.3390/ijms231810313

**Published:** 2022-09-07

**Authors:** Adri Chakraborty, Rachelle Crescenzi, Timaj A. Usman, Andrea J. Reyna, Maria E. Garza, Sara Al-Ghadban, Karen L. Herbst, Paula M. C. Donahue, Joseph M. Rutkowski

**Affiliations:** 1 Department of Medical Physiology, Texas A&M University School of Medicine, Bryan, TX 77807, USA; 2 Currently the Arthritis & Autoimmune Diseases Research Center, Boston University School of Medicine, Boston, MA 02118, USA; 3 Department of Radiology and Radiological Sciences, Vanderbilt University Medical Center, Nashville, TN 37232, USA; 4 Vanderbilt University Institute of Imaging Science, Vanderbilt University Medical Center, Nashville, TN 37232, USA; 5 Department of Biomedical Engineering, Vanderbilt University, Nashville, TN 37232, USA; 6 Department of Microbiology, Immunology & Genetics, University of North Texas Health Science Center, Fort Worth, TX 76107, USA; 7 Total Lipedema Care, Beverly Hills, CA 90211, USA; 8 Physical Medicine and Rehabilitation, Vanderbilt University Medical Center, Nashville, TN 37232, USA; 9 Dayani Center for Health and Wellness, Vanderbilt University Medical Center, Nashville, TN 37232, USA

**Keywords:** lipedema, neuropathy, adipose tissue, fat disorder, neuropeptide, dermis, pain

## Abstract

Lipedema is a disease with abnormally increased adipose tissue deposition and distribution. Pain sensations have been described in the clinical evaluation of lipedema, but its etiology remains poorly understood. We hypothesized that pain sensitivity measurements and ex vivo quantitation of neuronal cell body distribution in the skin would be lipedema stage-dependent, and could, thus, serve to objectively characterize neuropathic pain in lipedema. The pain was assessed by questionnaire and peripheral cutaneous mechanical sensitization (von-Frey) in lipedema (*n* = 27) and control (*n* = 23) consenting female volunteers. Dermal biopsies from (*n* = 11) Stages 1–3 lipedema and control (*n* = 10) participants were characterized for neuronal cell body and nociceptive neuropeptide calcitonin gene-related peptide (CGRP) and nerve growth factor (NGF) distribution. Stage 2 or 3 lipedema participants responded positively to von Frey sensitization in the calf and thigh, and Stage 3 participants also responded in the arm. Lipedema abdominal skin displayed reduced Tuj-1+ neuronal cell body density, compared to healthy controls, while CGRP and NGF was significantly elevated in Stage 3 lipedema tissues. Together, dermal neuronal cell body loss is consistent with hyper-sensitization in patients with lipedema. Further study of neuropathic pain in lipedema may elucidate underlying disease mechanisms and inform lipedema clinical management and treatment impact.

## 1. Introduction

Lipedema is a chronic loose connective tissue disease of abnormal subcutaneous adipose tissue deposition and distribution. Often misdiagnosed as general obesity, lipedema adipose is highly resistant to diet and exercise and patients exhibit a lower prevalence of metabolic syndrome than expected of individuals with elevated body mass index (BMI) [1]. Various reports and hypotheses suggest the etiology of lipedema’s aberrant adipose tissue expansion is related to vascular and lymphatic dysfunction, altered extracellular matrix composition, dysregulated hormones or metabolism, and potential genetic predisposition [2,3].

Lipedema patients commonly report allodynic and neuropathic pain in the affected tissues that is, importantly, unique from symptoms of obesity or lymphedema [4]. Pain is critical to address in the clinical management lipedema as over 90% of patients report some tissue pain [5]. Pain commonly affects the lower extremities with lipedema, with the affected areas increasing with the lipedema stage [2]. The underlying mechanisms of lipedema pain remain unknown, although disease pathology in affected areas, such as thighs and abdomen, are under investigation [6]. For example, imaging identifiers have correlated lower-extremity tissue sodium levels with lower-extremity pain in lipedema patients [7]. Reports of burning pain suggest that neurogenic inflammation triggered by sensory neurons may play an integral role in the sympathetic and parasympathetic regulation of pain in lipedema. These pathways have not been rigorously evaluated and there remains little understanding of associated lipedema pain correlates. Identifying and understanding factors involved in lipedema-associated pain could help accurately assess condition severity and may reveal mechanisms of lipedema pathogenesis.

Understanding the extent of or type of pain in lipedema has not been thoroughly characterized, with current reports predominantly relying on the quality of life assessments and pain surveys, such as the standard 10-point or 100-point visual analog scale (VAS) [8]. Other questionnaires have included Freiburg Quality of Life Assessment for lymphatic disorders, the six-minute walk test, patient-specific functional scale, and RAND-36 [4,9]. One finding is that lipedema-associated pain levels are independent of BMI and do not appear to be driven by inflammation per se [10]. Lipedema patient reports describe the pain in the affected tissue as oppressive, pressing, and pulling in nature, suggestive of mechanical allodynia or sensory neurosensitization [11].

In this investigation, we investigated the peripheral behavior of pain and dermal sensitivity as potential functional indicators of the hyperalgesia response within lipedema tissue, as well as the ex vivo assessment of neuronal cell body distribution and potential neuroinflammatory response in the skin, in a stage-dependent manner. Recruited subjects responded to the painDETECT survey regarding nociceptive pain and were tested with von Frey mechanical allodynia filaments to discern pressure-induced skin sensitivity in lipedema. Tissue biopsies from controls and patients with lipedema stages 1–3 were assessed for neuronal cell body distribution and nociceptive neuropeptides calcitonin gene-related peptide (CGRP) and nerve growth factor (NGF). Findings reveal that lipedema patients demonstrate nociceptive response to mechanical stimuli, changes in dermal neuronal cell body counts and inflammation in a stage-dependent manner.

## 2. Results

### 2.1. Lipedema Subjects Indicate Pain Symptoms as a Significant Factor in Their Disease

**Figure 1:**
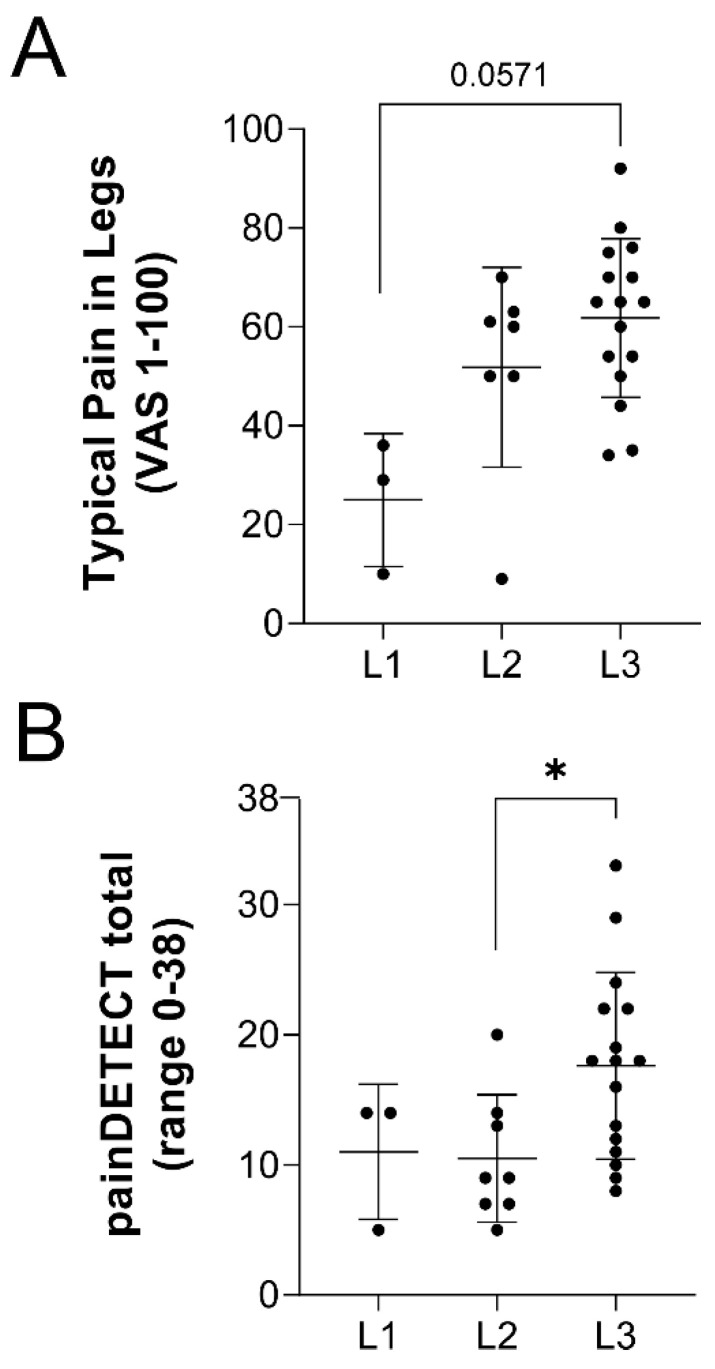
**VAS scores and painDETECT responses identify pain across lipedema stages.** (**A**) Responses on the visual analogue scale (VAS) in patients with lipedema regarding their typical leg pain. *p* = 0.0105 across stages by ANOVA. (**B**) Calculated painDETECT questionnaire scores across lipedema stage. *p* = 0.0271 across stages by ANOVA. Stage 1 *n* = 5, Stage 2 *n* = 13, Stage 3 *n* = 16. Mean ± SD. * indicates *p* < 0.05 by post hoc multiple comparison test.

The characteristics of participants assessed for pain using questionnaires and von Frey filaments are summarized in Table 1. The advanced lipedema phenotype in stage 3 patients was clear in significantly greater weight, BMI, waist and hip circumferences, and age. Of note, few participants were diabetic. Pain questionnaire responses are summarized in Table 2. Notably, all control subjects were null responders, while 60% of stage 1, 61.5% of stage 2, and 100% of stage 3 lipedema participants who completed the questionnaire were positive responders to having pain in their legs (Table 2 question (Q)1). Of these positive responders, a significant stage-wise increase in pain was identified by ANOVA between patients with lipedema, indicating pain increases with disease stage (*p* = 0.0105; Table 2 Q2, Figure 1A). A similar trend was observed with both the pain currently being experienced in the legs and the worst pain typically experienced in the legs, increasing with disease stage (Table 2 Q3, Q4). Mean cumulative painDETECT scores were significantly different across lipedema stages by ANOVA (*p* = 0.0271; Table 2 Q5, Figure 1B). Notably, stage 3 patients had significantly higher average scores and the only >19 (indicating neuropathic pain) respondent scores. Pain was described by participants to radiate from the legs to other areas of the body in 33.3% of lipedema stage 1, 37.5% of lipedema stage 2, and 62.5% of lipedema stage 3 participants. Differences in questionnaire score responses regarding the description of burning, tingling, shock sensation, or numbness were not significant across lipedema stages. Pain behavior patterns that were significantly different across lipedema stages were scores for light touching (*p* = 0.0122), temperature-induced pain (*p* = 0.0011), and pressure-induced pain (*p* = 0.0015) (Table 2 Q7). Again, stage 3 lipedema patients demonstrated the most significantly different scores, compared to stage 1 or 2 patients.

### 2.2. Cutaneous Mechanical Sensitivity Is Heightened in Lipedema

**Figure 2:**
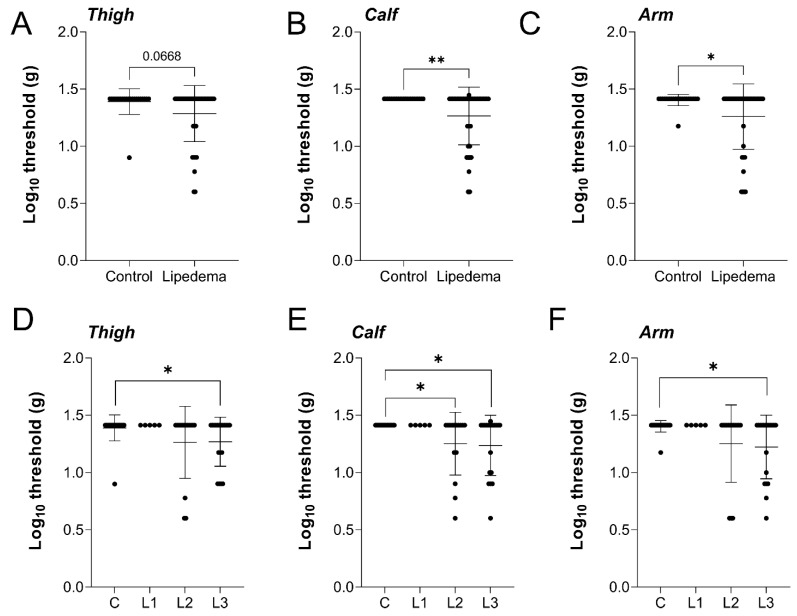
Mechanical von Frey sensitivity assay demonstrates heightened innocuous stimulation for Stage 2 and 3 lipedema patients. Lipedema vs. Control: representation of von Frey pain scores (as log10 fold increase pain intensity) tested in the right-side (**A**) upper lateral thigh, (**B**) anterior calf, and (**C**) upper lateral arm of the body. Lipedema stage-based representation of von Frey pain scores (as log10 fold increase pain intensity) tested in the (**D**) upper lateral thigh (*p* = 0.0772 across stages by ANOVA), (**E**) anterior calf (*p* = 0.0246 across stages by ANOVA), and (**F**) upper lateral arm (*p* = 0.0383 across stages by ANOVA) of the body. Control *n* = 23, Stage 1 *n* = 5, Stage 2 *n* = 13, Stage 3 *n* = 19. Mean ± SD. (**A**–**C**) * indicates *p* < 0.05, ** *p*< 0.01. (**D**–**F**) * indicates *p* < 0.05 by post hoc multiple comparison test.

Study participants were tested for pain-associated cutaneous sensitivity through von Frey mechanical stimulation on the thigh, calf, and arm. Despite the thigh being the most visually affected by lipedema, the mechanical sensory threshold only trended toward increased sensitivity but was not significantly different in lipedema patients, compared to controls (Figure 2A). In the calf and arm, however, lipedema patients were significantly more sensitive than controls (Figure 2B,C). When comparing across lipedema stage in the thigh, no overall stage effect in the cutaneous mechanical sensory threshold was identified (*p* = 0.0772), however, stage 3 lipedema patients were significantly more sensitive than controls (Figure 2D). A significant stage-dependent effect was measured in the calf (*p* = 0.0246) with significantly increased sensitization in both lipedema stage 2 and stage 3, compared to controls (Figure 2E). The arm response was similar across lipedema stage (*p* = 0.0383) with stage 3 lipedema demonstrating significant local hyperalgesia (Figure 2F). Overall, the objectively measured von Frey response indicated heightened dermal sensitivity in patients with lipedema, consistent with stage-dependent disease severity in this study cohort.

### 2.3. An Objective versus Subjective Comparison of Lipedema Pain

**Figure 3:**
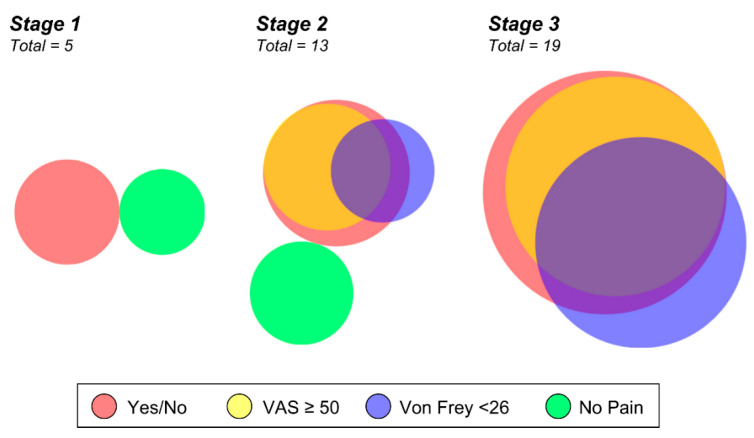
**Venn diagram of summarized pain assessments.** Proportional Venn Diagram of survey data illustrating lipedema subjects with a positive pain response to a simple yes or no survey (red), a VAS score greater than 50 (yellow, overlays as orange), a Von Frey threshold less than 26 (blue), and those without pain by the measures used (green). All controls reported no pain in their legs on yes/no questionnaire. Clusters are shown for participants with stage 1, 2, or 3 lipedema. Note that all patients with stage 3 lipedema reported pain in their legs (no green). Patients with stage 2 or 3 lipedema with pain in their legs rated the severity VAS > 50 (overlap between red and yellow), which is a clinically significant level. Some patients reported sensitivity to a filament-induced pain response who did not report pain in their legs or VAS > 50 (unique blue portion). Proportionality generated using DeepVenn (https://www.deepvenn.com, accessed on 22 February 2022).

Survey data illustrates what the individual patient is experiencing and is a clinically significant indicator that can guide treatment and therapy outcomes. Objective von Frey measurement is a functional assessment of pain, and we wanted to evaluate if this information would be redundant with the self-reported survey of pain or provide additional information for our cohort. In comparing the 27 lipedema patients by stage and by their responses to a simple “yes” or “no” regarding leg pain, their VAS score, and the von Frey result, both survey results largely overlapped (Figure 3). The objective von Frey measure did not wholly overlap with the survey results; it instead identified only some of the pain-reporting patients and interestingly identified some who did not self-report any peripheral neuropathic symptoms (Figure 3). Of note, 10 of 13 stage 2 lipedema patients had self-reported or measured aspects of pain, and all stage 3 participants were positive for pain assessment (19/19). These data further illustrate that pain is a significant part of lipedema’s clinical manifestation and increases with stage.

### 2.4. Neuronal Density Is Not Reduced in the Thigh but Is Reduced in the Abdominal Skin of Lipedema Patients

**Figure 4:**
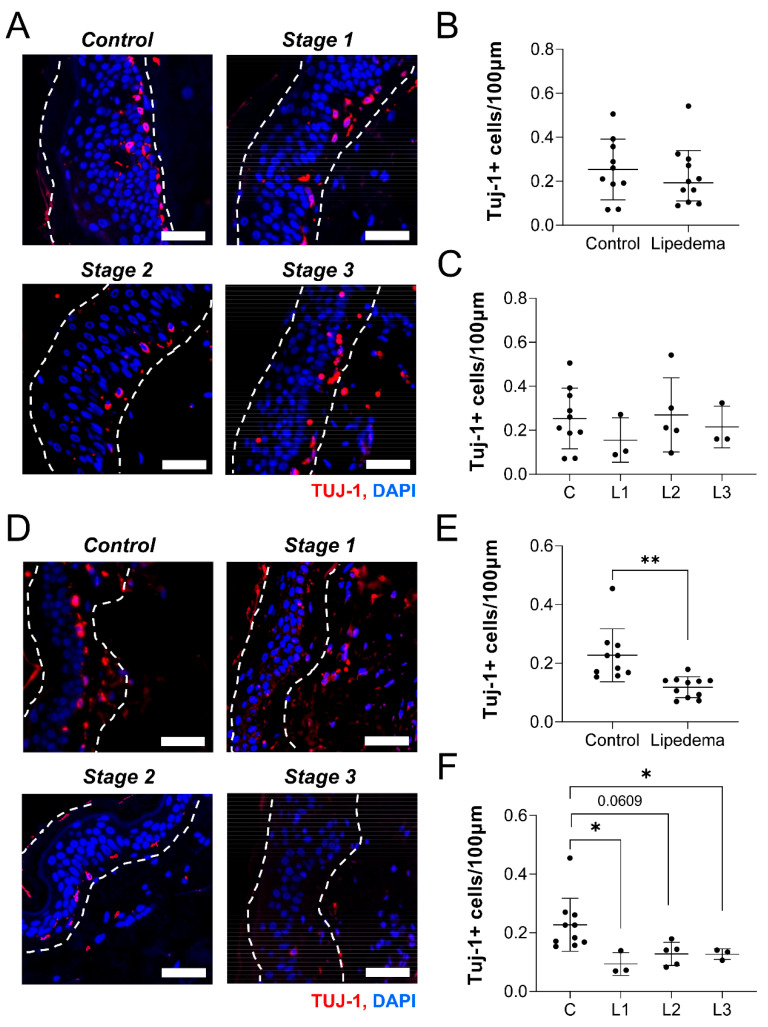
**Lipedema stage-based characterization of neuronal cell body distribution in thigh and abdomen dermal sections.** (**A**) Tuj1+ (red), nuclei (blue) in thigh dermis of non-lipedema control, 1, 2, and 3 lipedema. (**B**) Comparison between total Tuj1+ (red) cells per 100 nm length of the thigh dermis in control and total lipedema patients. (**C**) Comparison between total Tuj1+ (red) cells per 100 nm length of the thigh dermis in control and stage 1, 2, and 3 lipedema patients (*p* = 0.6161 across stages by ANOVA). (**D**) Tuj1+ (red), nuclei (blue) in abdominal dermis of non-lipedema control, and lipedema stages 1, 2, and 3. (**E**) Comparison between total Tuj1+ (red) cells per 100 nm length of the abdominal dermis in control and total lipedema patients. (**F**) Comparison between total Tuj1+ (red) cells per 100 nm length of the abdominal dermis in control and lipedema stages 1, 2, and 3 (*p* = 0.0011 across stages by ANOVA). (**A**–**F**) Control *n* = 10, Stage 1 *n* = 3, Stage 2 *n* = 5, Stage 3 *n* = 3. Bars = 100 µm. Mean ± SD. In (**E**), ** indicates *p* < 0.01; in (**F**) * indicates *p* < 0.05 by post hoc multiple comparison test.

The etiology of peripheral neuropathy involves reduced intradermal neuronal density [12,13]. We thus sought to identify changes in neuronal cell body density in the skin of patients with lipedema, compared to controls or between lipedema stage. A list of general participant characteristics is reported in Table 3 for the control (*n* = 10) and lipedema (*n* = 11) samples utilized. Only BMI was significantly different in the lipedema subjects, likely due to increased leg girth in Stage 3 participants. We counted the number of Tuj-1+ cell bodies per length of skin from thigh tissue biopsies and found no difference in density between control and lipedema samples (Figure 4A,B). There was also surprisingly no differences in cell body density across lipedema stage in the thigh biopsies (Figure 4C). Abdominal samples were originally thought to serve as pathology-null tissue control sites, but we identified a significant reduction in cell body density in patients with lipedema, compared to control participants (Figure 4D,E). This effect was significant across multiple stages of lipedema (*p* < 0.01; Figure 4F). Both Stage 1 and Stage 3 lipedema skin samples demonstrated a significant reduction in Tuj-1+ cell bodies, compared to control skin. Although it is interesting that total and stage-based distribution of Tuj-1+ cell bodies in the thigh did not change, we did observe a significant reduction in Tuj-1+ dermal neuronal body numbers between the control and lipedema stages in the abdomen. A reduction in Tuj-1+ dermal neuron density across lipedema stages in the abdomen is suggestive of a systemic changes associated with changes in lipedema tissue and neuronal density.

### 2.5. Lipedema Skin Exhibits Overexpression of Neuropeptides Assocatied with Neurogenic Inflammation

**Figure 5:**
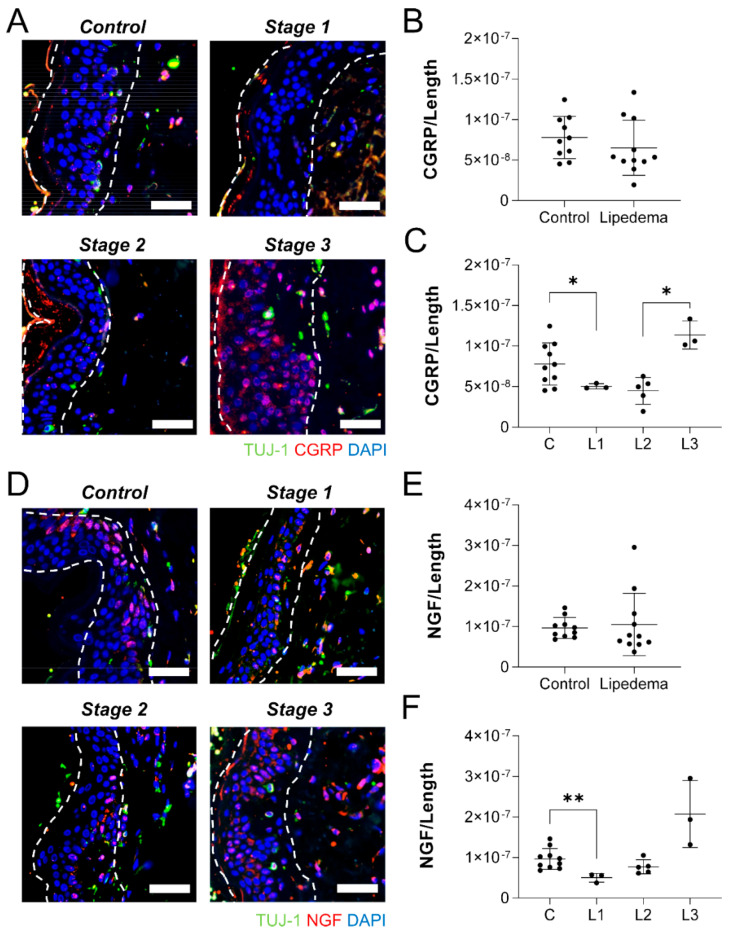
**Lipedema stage-based characterization of neuropathic markers in abdomen dermal sections.** (**A**) Tuj1+ (green), CGRP (red), and nuclei (blue) in abdominal dermis of stage 1, 2, and 3 lipedema. (**B**) CGRP fluorescence threshold normalized to the total length of the tissue in control and total lipedema patients. (**C**) CGRP fluorescence threshold normalized to the total length of the tissue in control and stage 1, 2, and 3 lipedema patients (*p* = 0.0108 across stages by ANOVA). (**D**) Tuj1+ (green), NGF (red), and nuclei (blue) in abdominal dermis of stage 0,1, 2, and 3 lipedema. (**E**) NGF fluorescence threshold normalized to the total length of the tissue in control and total lipedema patients. (**F**) NGF fluorescence threshold normalized to the total length of the tissue in control and stage 1, 2, and 3 lipedema patients (*p* = 0.0977 across stages by ANOVA). (**A**–**F**) Control *n* = 10, Stage 1 *n* = 3, Stage 2 *n* = 5, Stage 3 *n* = 3. Bars = 100 µm. Mean ± SD. * *p* < 0.05 and ** *p* < 0.01 by post hoc multiple comparison test.

With the dermal neuronal density reduced in lipedema abdominal, but not thigh samples, we sought to assess the tissues for other potential indicators of peripheral neuroinflammation. The immunolabeling of neuropathic markers CGRP and NGF was quantified across the length of the skin on tissue biopsies. No difference in dermal CGRP labeling was visualized or quantified in the abdominal skin lipedema samples, compared to controls (Figure 5A,B). When broken down by lipedema stage, however, a significant stage effect was identified (*p* < 0.01). Significantly less CGRP-positive labeling was measured in Stage 1 lipedema samples than in control tissue, while Stage 3 lipedema samples demonstrated a significant increase over Stage 2 skin (Figure 5C). NGF labeling results were very similar in the abdominal dermis, with no overall changes noted or quantified (Figure 5D,E). No stage-dependent effect was detected, however, Stage 1 samples has significantly less NGF cellular labeling than measured in control epidermis (Figure 5F).

**Figure 6:**
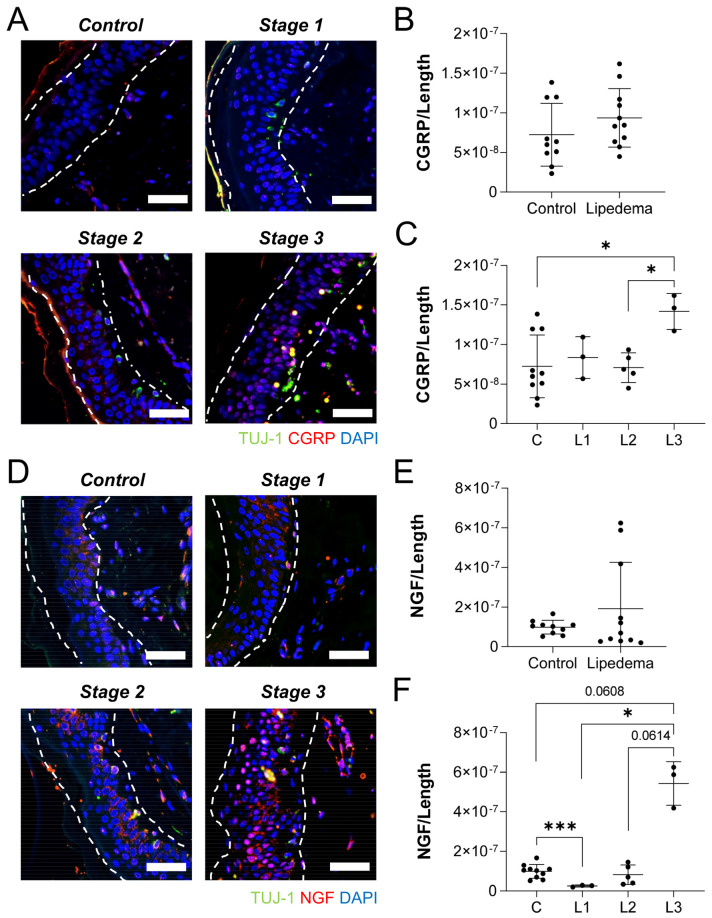
**Lipedema stage-based characterization of neuropathic markers in thigh dermal sections.** (**A**) Tuj1+ (green), CGRP (red), and nuclei (blue) in thigh dermis of stage 1, 2, and 3 lipedema. (**B**) CGRP fluorescence threshold normalized to the total length of the tissue in control and total lipedema patients. (**C**) CGRP fluorescence threshold normalized to the total length of the tissue in control and stage 1, 2, and 3 lipedema patients (*p* = 0.0108 across stages by ANOVA). (**D**) Tuj1+ (green), NGF (red), and nuclei (blue) in thigh dermis of stage 1, 2, and 3 lipedema. (**E**) NGF fluorescence threshold normalized to the total length of the tissue in control and total lipedema patients. (**F**) NGF fluorescence threshold normalized to the total length of the tissue in control and stage 1, 2, and 3 lipedema patients (*p* = 0.0054 across stages by ANOVA). (**A**–**F**) Control *n* = 10, Stage 1 *n* = 3, Stage 2 *n* = 5, Stage 3 *n* = 3. Bars = 100 µm. Mean ± SD. * *p* < 0.05 and ** *p* < 0.01 by post hoc multiple comparison test.

Conversely, a stronger stage dependence of CGRP and NGF immunolabeling was measured in the epidermis of thigh samples. Although total CGRP density (Figure 6A,B) was unchanged across lipedema tissue and controls, a stage-wise comparison showed a significant increase in CGRP expression between stage (*p* = 0.0108). Notably, CGRP labeling was significantly higher in stage 3 samples, compared to either control or stage 2 tissues (Figure 6C). Although no marked changes in the dermal NGF expression levels between overall lipedema and control samples were observed (Figure 6D,E), a stage-wise ANOVA identified a significant effect (*p* < 0.01; Figure 6F). Stage 1 samples had significantly less NGF density than controls, and NGF labeling in Stage 3 samples was higher than in any other stage or controls. Overall, our data suggest a consistently unique stage-dependent expression pattern of neuropeptides associated with neurogenic inflammation, which can be characterized in the epidermis of lipedema skin. The expression levels of NGF and CGRP were similar within abdomen and thigh across the stages, especially this effect, strongest in Stage 3 lipedema.

## 3. Discussion

While the underlying causes of lipedema remain largely unknown, it is clinically unique from general obesity and lower limb lymphedema [1,3]. Importantly, lipedema is different from these conditions because of the excessive pain experienced by women with lipedema in affected areas [11]. Here, we demonstrate that patients with lipedema consistently acknowledge and describe nociceptive pain, that this pain is measurable by an objective mechanical allodynia response, and that lipedema dermal tissue contains significant differences in neuronal cell body and neuropeptide distribution potentially driving neuronal sensitization.

Differential diagnosis of lipedema clinically indicates three disease stages (Stages 1–3) based on physical symptoms and subcutaneous fat distribution [1,5,14,15,16]; however, the common symptom of pain is not currently included in disease staging. This is, in part, because pain reports vary in their incidence and in the subjective survey methods that have been used in the limited number of clinical lipedema patient reports studying this aspect of the disease. Currently, a combination of comprehensive quality of life assessments, such as the 36-Item Short Form Survey (SF-36) that incorporates both pain and vitality, are used as clinical diagnostic tools to distinguish painful lipedema from other conditions; though pain diagnostics have largely not been studied across time and stages in patients with lipedema [4,17]. Early stage (stage 1) patients with lipedema often go undiagnosed, limiting sample sizes and available data (an admitted limitation of the current study as well). Furthermore, that lipedema symptoms are often confused with generalized obesity or Dercum’s disease (a distinct and well-characterized disorder of painful lipoma growth) only adds to the confusion regarding any indicated and type of ‘pain’ [18]. Descriptors of lipedema pain have often included pressure-induced pain, burning sensation, tingling, and numbness that each adversely affects the quality of life in patients with lipedema [11,19]. In this study, diagnostic tools were explored to identify and objectively categorize pain, including dermal sensitivity in patients with lipedema. Survey results from a VAS score and painDETECT questionnaires with positive pain findings overlapped and our lipedema volunteers largely confirmed temperature and touch- or pressure-induced pain. Functional von Frey assessments provided evidence that lipedema pain can be quantified by objective, mechanical stimulation to the skin. Surprisingly, mechanical sensitization was strongest in the calf and arm of lipedema patients despite thighs displaying the greatest SAT expansion. In patients with stage 2 or 3 lipedema, compared to controls, significant hypersensitization of the dermis in all affected areas was demonstrated; thus, confirming patients’ survey responses. Pain measurement by von Frey to objectively categorize pain could be used to improve the diagnosis of this disease, help assess treatment outcomes, and hint at the underlying pathogenesis of this condition.

Like much of the etiology of lipedema, what drives the pain phenotype is not entirely clear. Some hypotheses have attributed this to generic adipose tissue inflammation [10]. Other factors, such as dysregulated adipogenesis, adipose macrophage recruitment, serum vascular endothelial growth factor-c (VEGF-C) increase, and blood and lymphatic vascular integrity, have also been proposed to contribute to lipedema, but their impact on altered nociception is also yet to be established [6,20]. A recent genetic study identifying a mutation in *Akr1c1* in a family with lipedema speculated that the analgesic activity of allopregnanolone may play a role in lipedema-associated pain [21]. In this study, we identified the expression of two key neuropeptides (CGRP and NGF) that have been previously associated with nociceptive pain pathways or neurogenic inflammation are significantly altered in the skin of lipedema patients–lower in early stages and raised in Stage 3 participants, compared to controls [22]. We also show significantly decreased neuron body/subcutaneous neuronal precursor density (neuron-specific class III β-tubulin Tuj-1) in lipedema abdominal skin. These findings are similar to those found for diabetic peripheral neuropathy wherein significantly reduced Tuj-1 levels and nerve fibers were reported in those with the most pain [12,13]. CGRP expression being reduced in some patient samples was similar to findings in painful diabetic neuropathy as well [22]. Importantly, findings such as reduced nerve fiber density were independent of BMI, supporting our data that it is not merely an “expansion” of the tissue in lipedema causing reduced Tuj-1 cell density [23]. Neurogenic inflammation is often mediated by an acute sensitization of sensory neurons in response to released neuropeptides, such as NGF, and impacts vasculature remodeling, immune cell extravasation, and tissue inflammation [22,24]. Chronic sensitization of peripheral nerve endings hypersensitizes the neurons resulting in autologous neurotransmitter release (CGRP and Substance *p*) contributing to neurogenic inflammation [25,26,27]. It is also possible that the altered CGRP or NGF expression identified in lipedema skin may result from underlying tissue inflammation driven by other cytokines not measured in this study. Which one occurs first in relation to vascular, immunologic, and inflammatory markers in lipedema, should be further studied to better understand the unique etiology of pain in lipedema.

Finally, it is worth noting that there may be a relationship between peripheral and central nervous system involvement in lipedema pain that deserves further research. The psychological nature of pain in lipedema has been suggested to involve central sensitization [28]. In fact, imaging of the cerebrovascular system in lipedema revealed significantly elevated cerebral blood flow in patients with lipedema who also reported significant peripheral pain [29]. Our findings of significant changes in dermal sensitivity and neuronal markers in areas with less pronounced fat expansion support the possibility that lipedema is a systemic condition. Improved understanding of the systemic vs. local nature of lipedema’s pathogenesis should be explored in further studies.

## 4. Materials and Methods

### 4.1. Human Subject Recruitment

Study participants who were evaluated for questionnaires and functional pain response were in attendance at the Fat Disorders Resource Society (FDRS) annual conference (2019, Baltimore, MD, USA) or recruited from the local community at the time of the meeting using ResearchMatch.org [30]. All control (*n* = 23) and lipedema (*n* = 27) subjects (summarized in Table 1) participating in the study provided informed consent in accordance with the Vanderbilt University Medical Center Institutional Review Board (protocol 160199).

### 4.2. Clinical Examination and Pain Questionnaires

A physical exam was performed by a clinician with 13 years of specialization in lipedema and in determining lipedema stage. Participants with lipedema met all primary criteria (bilateral swelling leg swelling, a negative Stemmer’s sign, and positive for lipedema by stage and type) and at least two secondary criteria (lower extremity pain, family history of lipedema, joint hypermobility, or easy leg bruising) as previously described [31]. Lipedema staging followed the criteria recently described in a lipedema “standard of care” manuscript [1,32]. During the physical exam, height and weight were measured and converted to metric units and BMI calculated (kg/m^2^). Shoulder, waist, and hip circumference were measured and waist-to-hip ratio (WHR) calculated.

Participants also completed electronic self-administered surveys through REDCap™ regarding symptoms of lipedema that included general questions about pain.

Specifically, the presence or absence of pain in the lower extremities was inquired about. Positive responders were further inquired about the level of pain experienced in their legs on the visual analogue scale (VAS, range 0–100) (Supplemental Material File S1) and completed painDETECT questions regarding the behavior and degree of neuropathic pain in their legs (Supplementary Material File S2). Pain behavior patterns were rated never (0) to very strongly (5). A cumulative pain score was calculated for painDETECT reflective of degree of neuropathic pain (possible score range = 0 to 38); values > 19 indicate neuropathic pain positive, values 13–18 indicate neuropathic pain unclear, and values < 13 indicate neuropathic pain negative.

### 4.3. Von Frey Pain Assessment

To obtain a quantitative measure of allodynic pain, von Frey filaments were applied to measure skin sensitivity threshold in response to a mechanical stimulus [33]. Sensory thresholds vary for human participants in the arms and legs, and typically range from 1.57–9.81 milliNewtons (mN) applied by a plastic filament. The trials were performed by a standard experimenter using von Frey monofilaments (Bioseb US, Pinellas Park, FL, USA) [33]. Before the start of the first experiment, the filaments were manipulated by gently bending them against the skin of the experimenter’s hand. Enrolled participants were asked to sit on the side of a bed, with support provided for the extremity to be tested. Filaments with force range 0.078 mN (0.008 g) to 4.08 mN (1.0 g) were applied on the right side of the body at one control site (palm), and three test sites: anterior calf, lateral thigh, and back of upper arm. Four positive and negative trials at each site were conducted. For a positive trial, the filament was applied to the site and the subject asked if they could feel the filament; during a negative trial, the same question was asked although the filament was *not* applied. During each trial, the subject was asked to look away from the site of application. Trials were started using the smallest filament. For each filament, if the subject detected <2 positive trials and/or >0 negative trials, another round of trials with the next highest pressure was started. When the subject detected ≥3 positive trials and 0 negative trials for a filament, the subject’s “mechanical sensory threshold” was reached and the filament strength recorded for that site. All participants felt the highest force filament on the palm, which was a control site and force for the test (i.e., to make sure their body could feel strong filaments at a sensitive site). The mechanical sensory threshold was calculated as the log_10_ fold difference, compared to the threshold at the palm, such that lower thresholds indicate higher sensitivity.

### 4.4. Histology and Immunofluorescence

Existing paraffin-embedded tissue sections from abdominal and anterolateral thigh dermal punch biopsies from lipedema patients (*n* = 11) and controls (*n* = 10) were obtained [6]. Specific inclusion and exclusion criteria for these patients has been previously described [6]. Paraffin-embedded tissue sections 0.5 µm thick were deparaffinized and labeled with primary antibodies against anti-ß-tubulin III (Tuj-1) (ab18207; Abcam, Waltham, MA, USA), calcitonin gene-related peptide (CGRP) (ab272713; Abcam), and nerve growth factor (NGF) (ab52918; Abcam), with fluorophore-conjugated secondary detection.

### 4.5. Image Analysis

Immunofluorescence was imaged using an Olympus VS120 virtual slide scanner system in the Texas A&M Health Science Center Integrated Microscopy and Imaging Laboratory Core Facility (RRID:SCR_021637). Scans were captured under 10× magnification with equal exposure settings for all images of the same label. All the analysis was performed using ImageJ software version 1.52 (NIH, Bethesda, Rockville, MD, USA). Positive Tuj-1 immunolabeled cell bodies were manually counted and normalized to 100 nm length of the dermis represented as Tuj-1 nuclei/100 nm skin. CGRP and NGF were quantified as the positive area exceeding a fixed fluorescence threshold using Costes colocalization near a DAPI signal normalized to the total length of the tissue. The width (thickness) of the epidermis was similar across samples and is indicated in the figures. The stratum corneum was excluded from the analysis area.

### 4.6. Statistical Analysis and Data Presentation

For histological studies, minimally three tissue sections per lipedema stage were used. All statistical tests were performed in Prism software (v. 9.3.0; GraphPad Software, La Jolla, CA, USA). Von Frey results were not parametric, so to compare controls versus lipedema, a Mann–Whitney test was utilized. When comparing von Frey results across stage, a non-parametric Kruskal–Wallis test was performed with a Benjamin, Krieger, and Yekutieli correction for multiple comparisons to control for the false discovery rate. Pain survey data and histological data were largely parametric (or the *n* was small), so parametric testing was used with a Welch’s corrected (unequal SDs) *t*-test utilized for overall lipedema versus controls. ANOVA was used to compare across stages using the Brown-Forsythe ANOVA and Dunnett T3 correction for multiple comparisons (for *n* < 50). All data are presented as means ± SD. *p* < 0.05 was considered significant, though trends are reported if *p* < 0.07.

## 5. Conclusions

There is a growing clinical consensus that therapeutic management of lipedema should consider the severity and location of pain present with disease severity. For instance, patients report a reduction in surveyed pain with use of compression garments or following limb volume reduction [1,34,35]. Though few treatment options specifically addressing pain are in use, a diagnostic approach to clinically identify pain in lipedema could objectively test and evaluate potential treatment options for one of the most troubling symptoms of lipedema. The changes in neuron density or indications of neurogenic inflammation identified here also support underlying biological changes and potential inflammation in the tissue of lipedema patients. Together, the assessment of the neuronal profile and functional sensitivity of the dermis in lipedema utilized in this study could assist with therapeutic research.

## 6. Summary

Lipedema-associated peripheral pain is validated through pain surveys, allodynia testing, and neuronal markers through patient biopsies demonstrating disease stage-dependent neuropathic pain, reduced neuronal density, and altered neuropeptide expression.

## Figures and Tables

**Table 1:** **Physical characteristics of participants assessed for pain and von Frey sensitivity.**

	Controls (*n* = 23)	Stage 1 (*n* = 5)	Stage 2 (*n* = 13)	Stage 3 (*n* = 19)	ANOVA *p*-Value
Age (years)	39.7 ± 16.8	41.4 ± 11.0	50.5 ± 10.1	57.5 ± 8.5 ^a,b^	*0.0002*
BMI (kg/m^2^)	26.6 ± 5.4	29.3 ± 7.0	32.1 ± 7.6	42.2 ± 6.7 ^a,b,c^	*<0.0001*
Shoulder circumference (cm)	108.3 ± 12.7	110.3 ± 11.6	110.3 ± 11.1	118.8 ± 11.5	*0.0456*
Waist circumference (cm)	84.7 ± 14.7	88.4 ± 17.6	91.4 ± 16.4	109.7 ± 14.1 ^a,c^	*0.0005*
Hip circumference (cm)	106.0 ± 12.7	110.7 ± 14.7	121.8 ± 18.3 ^a^	141.5 ± 18.4 ^a,b,c^	*<0.0001*
WHR	0.80 ± 0.08	0.79 ± 0.06	0.75 ± 0.10	0.78 ± 0.09	0.5559
Diabetes	4.3% (*n* = 1/23)	0%	23% (*n* = 3/13)	0%	n/a

BMI: body mass index; WHR: waist-to-hip ratio. Stage-dependent listed *p*-value calculated by ANOVA. ^a^ indicates *p* < 0.05 compared to control, ^b^ indicates *p* < 0.05 compared to stage 1, ^c^ indicates *p* < 0.05, compared to stage 2 by post hoc multiple comparison testing.

**Table 2:** **Summarized pain questionnaire responses questions.** Questions 1–4 are regarding leg pain by VAS response, while 5–7 are taken from the painDETECT survey questions regarding nociceptive pain descriptions. All controls responded “no” to Question 1. All subsequent questions were only inquired of those who responded “yes” to Question 1.

	Stage 1	Stage 2	Stage 3	ANOVA *p*-Value
1. Pain in legs (%yes, *n* = number of positive responders/total subjects)	60.0% (*n* = 3/5)	61.5% (*n* = 8/13)	100% (*n* = 16/16)	n/a
2. Typical pain in legs (VAS, 0–100)	25.0 ± 13.5	51.9 ± 20.2	61.8 ± 16.0	*0.0105*
3. Pain now in legs (VAS, 0–100)	19.7 ± 16.2	38.1 ± 25.4	53.4 ± 25.6	0.0580
4. Worst pain in legs in past 4 weeks (VAS, 0–100)	55.7 ± 20.8	69.1 ± 21.9	79.2 ± 12.1	0.2050
5. painDETECT: cumulative score (range 0–38)	11.0 ± 5.2	10.5 ± 4.9	17.6 ± 7.2 ^b^	*0.0271*
6. painDETECT: Does your pain radiate in your legs or to other areas of your body? (%yes)	33.3%	37.5%	62.5%	n/a
7. painDETECT: pain behavior patterns rated never (0) to very strongly (5) (mean ± standard deviation)	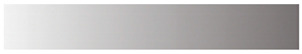 never (0) very strongly (5)			
a. Do you suffer from a burning sensation (e.g., stinging needles) in the marked areas?	1.7 ± 1.5	1.6 ± 1.5	2.4 ± 1.3	0.9693
b. Do you have a tingling or prickling sensation in the area of your pain (like crawling ants or electrical tingling)?	2.0 ± 1.0	1.8 ± 1.9	2.1 ± 1.6	0.8908
c. Is light touching (clothing, a blanket) in this area painful?	0.33 ± 0.1	0.6 ± 0.9	1.4 ± 1.3 ^a^	*0.0122*
d. Do you have sudden pain attacks in the area of your pain, like electric shocks?	1.0 ± 1.0	1.1 ± 1.2	2.4 ± 1.5	0.0532
e. Is cold or heat (bath water) in this area occasionally painful?	0.33 ± 0.1	0.8 ± 0.9	1.9 ± 1.4 ^a^	*0.0011*
f. Do you suffer from a sensation of numbness in the painful areas?	2.0 ± 2.0	1.8 ± 0.7	2.3 ± 1.6	0.7753
g. Does slight pressure in this area trigger pain?	2.7 ± 0.6	1.9 ± 0.6	3.6 ± 1.7 ^b^	*0.0015*

Stage-dependent listed *p*-value calculated by ANOVA. ^a^ indicates *p* < 0.05 compared to stage 1, ^b^ indicates *p* < 0.05 compared to stage 2 by post hoc multiple comparison testing.

**Table 3:** **Demographics for cohorts of human tissue samples.**

Characteristic	Control	Lipedema
Number of subjects	10	11
Age (years)	41.3 ± 10.6	43.8 ± 10.6
BMI (kg/m^2^)	26.0 ± 5.7	31.8 ± 4.3 *
Sex (% female)	100 %	100%
Race (% White)	90% (*n* = 9/10)	100%
Ethnicity (% Hispanic)	30% (*n* = 3/10)	0%
Menopausal status (% pre-menopausal)	70% (*n* = 7/10)	64% (*n* = 7/11)

* *p* < 0.05 compared to controls.

## Data Availability

Data from this study are available from the researchers upon reasonable request.

## Supplementary Materials

The following supporting information can be downloaded at: https://www.mdpi.com/article/10.3390/ijms231810313/s1.
