# Supplementary material for: Indications of Peripheral Pain, Dermal Hypersensitivity, and Neurogenic Inflammation in Patients with Lipedema
Source: Int J Mol Sci. 2022 Sep 7;23(18):10313. doi: 10.3390/ijms231810313 (PMC9499469; doi:10.3390/ijms231810313)
Supplement: Supplementary file 1 [file ijms-23-10313-s001.zip › Supplement 1 Survey.pdf]

Research 2019 Symptom Survey

Thank you for your interest in our Vanderbilt Lipedema research. We greatly appreciate your support. If you have any questions, please contact us at INFORM@vumc.org.

Thank you!

First name	____________________
Last Name	____________________
Phone Number	____________________
Email	____________________
Sex	◯ Female<br>◯ Male
Age	____________________<br>(Enrolling women ages 18 years and older)
Please enter your Height in total inches	____________________<br>(inches)
Please enter your Weight in pounds	____________________<br>(lbs.)
Have you gone through menopause?	◯ Yes<br>◯ No
Approximately, when was the first day of your most recent period?	____________________<br>(Date (D-M-Y))
At what age did you go through menopause?	____________________

Do you have a history of:

	Yes	No
Hypertension	☐	☐
Ischemic Heart Disease	☐	☐
Diabetes	☐	☐
Neurological Condition (i.e. Parkinson's, MS, Epilepsy, Alzheimer's, Central Nervous System Disease, Demyelinating Disease)	☐	☐
Ehlers-Danlos Syndrome	☐	☐
High Cholesterol	☐	☐
Stroke	☐	☐
Headaches	☐	☐
Possible Thyroid Problems	☐	☐
Lupus	☐	☐
Rheumatoid Arthritis	☐	☐
Other Autoimmune Diseases	☐	☐
Dercum's Disease	☐	☐
Clumsy or Uncoordinated	☐	☐
Mood swings	☐	☐
Depression	☐	☐
Hypermobility	☐	☐
Cancer	☐	☐
Chronic Venous Insufficiency (CVI)	☐	☐

Please list the autoimmune diseases do you have? ______________________________

For Diabetes, do you have Type 1 (childhood) or Type 2 (adult onset)?

- ◯ Type 1 (childhood)
- ◯ Type 2 (adult onset)
- ◯ Not sure

For thyroid problems, which do you have?

- ◯ hyperthyroid
- ◯ hypothyroid
- ◯ my doctor thought I may have thyroid issues and everything came back normal
- ◯ not sure
- ◯ no issues with my thyroid that I know of

What cancer have you been treated for? ______________________________

Do you take any medications (prescription or over-the-counter) or supplements? List the brand name and how often you take them (daily, weekly, every so often). ______________________________

What surgeries have you had since birth?

____

Do you know or think you have lipedema?

◯ Yes
◯ No
◯ Not Sure

How did you figure out you had lipedema?

◯ Physician's diagnosis
◯ Physical or lymphedema therapist
◯ Nurse Practitioner
◯ Friend/ Family member
◯ Self
◯ Internet/ Social Media
◯ Other

What is the way you figured out or think you have lipedema?

____

If so, tell us the physician's name who diagnosed you with Lipedema:

____

How long has it been since your diagnosis of lipedema?

◯ Less than 1 year
◯ 1-2 years
◯ 3-5 years
◯ 5-10 years
◯ Not sure

How long has it been since you suspected having lipedema?

◯ Less than 1 year
◯ 1-2 years
◯ 3-5 years
◯ 5-10 years
◯ Not sure

Lipedema has different stages. The following information is taken from Fat Disorders Research Society (FDRS) website: http://fatdisorders.org/fat-disorders/lipedema-lipoedema-description.

Stages of Lipedema

Stage I
1. Skin is smooth
2. Swelling increases during the day and may resolve with rest and elevation
3. Responds well to treatment

Stage II
1. Skin has indentations
2. Lipomas may develop
3. Eczema and erysipelas may be present
4. Swelling increases during the day, with less resolution after rest and elevation
5. May respond well to treatment

Stage III
1. Hardened connective tissue/fibrosclerosis
2. Swelling consistently present
3. Large masses of skin and fat that overhang
4. Less responsive to some treatment modalities

Stage IV
1. Fibrosclerosis, possibly elephantiasis
2. Swelling consistently present
3. Larger masses of skin and fat that overhang
4. Also known as Lipo-Lymphedema
5. Less responsive to some treatment modalities

Do you have a family history of Lipedema?	◯ Probably<br>◯ Probably Not<br>◯ Not Sure
What stage of Lipedema do you currently have? The above information describes the stages of Lipedema.	☐ Stage 1<br>☐ Stage 2<br>☐ Stage 3<br>☐ Stage 4<br>☐ Not sure

In addition to Lipedema Stages, there are different types of Lipedema. Below is information about the types of lipedema

In addition to stages of lipedema, there are types of lipedema. The type of lipedema is based on the distribution of fat.<br><br>What type of Lipedema do you currently have? Check all that apply.<br><br>Please Note: Type IV may occur with other types. Check all that apply.	☐ Type I: buttock and hips<br>☐ Type II: buttock to knees<br>☐ Type III: buttock to ankles<br>☐ Type IV: arms and legs affected<br>☐ Type V: lipolymphedema (lymphedema in addition to lipedema)<br>☐ Not sure
Approximately what age did lipedema symptoms start for you?	________________________<br>(age when symptoms started)
Do you think you have Lymphedema (swelling) of the legs and/or lower body?	◯ Yes<br>◯ No<br>◯ Sometimes (can come and go)<br>◯ Not Sure
Is your lymphedema because of cancer treatment?(i.e. radiation and/or lymph node removal)	◯ Yes<br>◯ No
Did you have lymph nodes removed?	◯ Yes<br>◯ No
Where in your body were lymph nodes removed and about how many were removed?	______________________________
Did you have radiation treatment?	◯ Yes<br>◯ No
How did you figure out that you had lymphedema?	◯ Physician's diagnosis<br>◯ Physical or lymphedema therapist<br>◯ Nurse pratictioner<br>◯ Internet<br>◯ Other
If so, tell us the physician's name who diagnosed you with Lymphedema:	______________________________

How long has it been since your diagnosis of Lymphedema?	◯ Less than 1 year<br>◯ 1-2 years<br>◯ 3-5 years<br>◯ 5-10 years<br>◯ Not sure
Please mark all the body areas you feel you have lymphedema	☐ right arm<br>☐ left arm<br>☐ right leg<br>☐ left leg<br>☐ torso<br>☐ head/neck
What Stage of Lymphedema do you currently have?<br><br>Stage 0<br>-A person is at risk for lymphedema but no visible signs of swelling are present<br><br>Stage I<br>-Visible swelling where elevation of the limb can temporarily help<br>-Swollen tissues are soft and pitting (indentations of skin when pushed on)<br><br>Stage II<br>-Increase in swelling and changes to the feel of the tissue<br>-Elevation of the limb will no longer reduce the swelling<br>-The tissues become increasing firm to touch<br>-Pressure against the limb produces only a slight indentation if any<br><br>Stage III<br>-Further progression of skin changes and swelling<br>-The tissues become increasingly fibrotic (hardened).<br>-Pressure on the skin does not produce any pitting (indentations)<br>-The skin may change color (darken) and feel thick and leathery<br>-Infections become more common	☐ Stage 0<br>☐ Stage 1<br>☐ Stage 2<br>☐ Stage 3<br>☐ Not sure
Lymphedema in my legs developed as a result of a cancer related surgery or radiation treatment	◯ Yes<br>◯ No<br>◯ Not sure
Approximately what age did lymphedema symptoms start for you?	______________________________<br>(years)

Please check symptoms (if any) you experience related to lipedema or lymphedema.	☐ None<br>☐ Easy bruising<br>☐ Urine odor<br>☐ Attractive to mosquitos<br>☐ Vericose veins<br>☐ Swell more in the affected areas in hot weather<br>☐ Altered walking (gait)<br>☐ Hypermobility of the joints (very flexible)<br>☐ Fatigue<br>☐ Hyperactivity<br>☐ POTS (Postural Orthostatic Tachycardia Syndrome)<br>☐ Flu-like symptoms (when you don't have the flu)<br>☐ Lipoma (soft tissue nodule or fatty mass under the skin)<br>☐ Skin of affected areas cold to the touch<br>☐ Skin of affected areas more pale in color<br>☐ Affected areas have fat that feels soft and lumpy (like cellulite)<br>☐ Hands and feet do NOT appear to be affected<br>☐ Skin and fat around the knee joints feels loose and floppy
How often do you experience fatigue?	☐ Daily<br>☐ Bi-weekly<br>☐ Weekly<br>☐ Monthly<br>☐ Every once in a while
Have any of these hormonal-events triggered or changed your symptoms of lipedema or lymphedema?	☐ Puberty<br>☐ Menstruation<br>☐ Pregnancy<br>☐ Menopause<br>☐ Abdominal surgery triggered my symptoms, such as cesarean section, hysterectomy, or other<br>☐ I think hormones affect my symptoms but not sure when<br>☐ I do not think hormones change my symptoms
Describe other symptoms you experience related to Lipedema OR Lymphedema:	__________________________________

Check all appropriate boxes to describe each part of your body. Indicate "Normal" if you are not concerned about this part of your body. Indicate "Not Symmetric" if enlargement is different between Right and Left sides of your body.

	Normal	Not Sure	Excess Fat Deposits (nodules)	Swelling (edema, excess fluid)	Painful	Not Symmetric
Calves	☐	☐	☐	☐	☐	☐
Knees	☐	☐	☐	☐	☐	☐
Thighs	☐	☐	☐	☐	☐	☐
Feet	☐	☐	☐	☐	☐	☐
Hips	☐	☐	☐	☐	☐	☐
Waist	☐	☐	☐	☐	☐	☐
Upper Arms	☐	☐	☐	☐	☐	☐
Forearms	☐	☐	☐	☐	☐	☐
Hands	☐	☐	☐	☐	☐	☐

Do you tend to have pain in your legs? ◯ Yes ◯ No

Choose the level of pain you normally experience in your legs on a typical day:

No Pain — Hurts — Worst Pain

(Place a mark on the scale above)

How would you assess your pain NOW, at this moment in your legs?

No pain — Hurts — Worst pain

(Place a mark on the scale above)

How strong was the strongest pain in your legs during the past 4 weeks?

No pain — Hurts — Worst pain

(Place a mark on the scale above)

Mark the phrase that best describes the course of your pain in your legs:

◯ Persistent pain with slight fluctuations
◯ Persistent pain with pain attacks
◯ Pain attacks without pain between them
◯ Pain attacks with pain between them

Does your pain radiate in your legs or to other areas of your body? ◯ Yes ◯ No

Answer the following questions regarding pain in your legs.

	Never	Hardly noticed	Slightly	Moderately	Strongly	Very strongly
Do you suffer from a burning sensation (e.g. stinging needles) in the marked areas?	○	○	○	○	○	○
Do you have a tingling or prickling sensation in the area of your pain (like crawling ants or electrical tingling)?	○	○	○	○	○	○
Is light touching (clothing, a blanket) in this area painful?	○	○	○	○	○	○
Do you have sudden pain attacks in the area of your pain, like electric shocks?	○	○	○	○	○	○
Is cold or heat (bath water) in this area occasionally painful?	○	○	○	○	○	○
Do you suffer from a sensation of numbness in the painful areas?	○	○	○	○	○	○
Does slight pressure in this area trigger pain?	○	○	○	○	○	○

Check any treatments that you have tried to modify symptoms of lipedema or lymphedema.

- ☐ Diet
- ☐ Exercise
- ☐ Supplements
- ☐ Liposuction
- ☐ Gastric Bypass Surgery
- ☐ Manual Lymphatic Drainage
- ☐ Compression garments
- ☐ Other
- ☐ None

How often do you receive Manual Lymphatic Drainage?

- ○ 2+ times a week
- ○ weekly
- ○ Twice a month
- ○ Monthly
- ○ Every 3-6 months
- ○ Yearly
- ○ Other

Has Manual Lymphatic Drainage been helpful at combating your symptoms?

- ○ Yes
- ○ No
- ○ Partially
- ○ Not Sure

Was diet or exercise effective in reducing any of your symptoms?

- ○ Yes
- ○ No
- ○ Partially
- ○ Not Sure

What part of your body received liposuction?

- ☐ Face
- ☐ Arms
- ☐ Stomach
- ☐ Buttocks
- ☐ Thighs
- ☐ Calves
- ☐ Other

What other body area underwent liposuction? ______________________

When did you receive liposuction? If possible, list the treating physician or surgery center. ______________________

Do you feel that liposuction has helped your symptoms?
- ◯ Yes
- ◯ No
- ◯ Partially
- ◯ Not Sure

When did you receive Gastric Bypass surgery? If possible, list the treating physician and the surgery center. ______________________

Do you feel that Gastric Bypass surgery has helped your symptoms?
- ◯ Yes
- ◯ No
- ◯ Partially
- ◯ Not Sure

What type of compression garments do you wear?
- ☐ Day-time
- ☐ Night-time

Where on your body do you wear compression garments? Check all that apply.
- ☐ Arm
- ☐ Hand
- ☐ Torso
- ☐ Full leg
- ☐ Knee high only

Describe other treatments you have tried for Lipedema OR Lymphedema, and whether you think they were effective or not. ______________________

Would you like to be contacted for FUTURE Lipedema or Lymphedema studies not necessarily related to the FDRS conference? This will never obligate you to participate in the future.
- ◯ Yes
- ◯ No

Paper Survey
