# Supplementary material for: Indications of Peripheral Pain, Dermal Hypersensitivity, and Neurogenic Inflammation in Patients with Lipedema
Source: Int J Mol Sci. 2022 Sep 7;23(18):10313. doi: 10.3390/ijms231810313 (PMC9499469; doi:10.3390/ijms231810313)
Supplement: Supplementary file 1 [file ijms-23-10313-s001.zip › Supplement 2 painDETECT.pdf]

painDETECT

PAIN QUESTIONNAIRE

Date: ------------------------ **Patient:** **Last name:** ---------------------------------- **First name:** --

How would you assess your pain **now**, at this moment?

0	1	2	3	4	5	6	7	8	9	10

none max.

How strong was the **strongest** pain during the past 4 weeks?

0	1	2	3	4	5	6	7	8	9	10

none max.

How strong was the pain during the past 4 weeks **on average**?

0	1	2	3	4	5	6	7	8	9	10

none max.

Mark the picture that best describes the course of your pain:

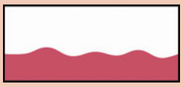
Persistent pain with slight fluctuations ☐

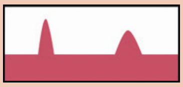
Persistent pain with pain attacks ☐

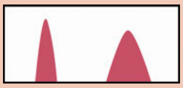
Pain attacks without pain between them ☐

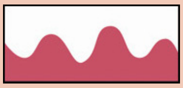
Pain attacks with pain between them ☐

Please mark your **main area of pain**

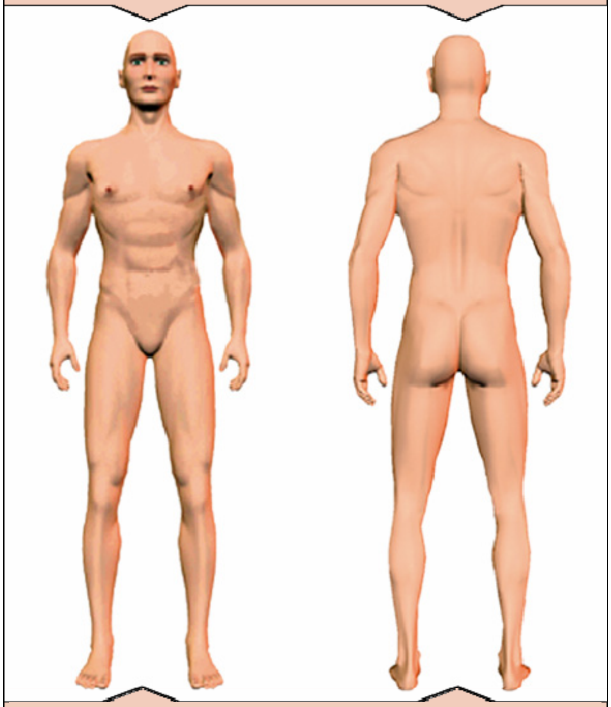

Does your pain radiate to other regions of your body? yes ☐ no ☐

If yes, please draw the direction in which the pain radiates.

Do you suffer from a burning sensation (e.g., stinging nettles) in the marked areas?

never ☐ hardly noticed ☐ slightly ☐ moderately ☐ strongly ☐ very strongly ☐

Do you have a tingling or prickling sensation in the area of your pain (like crawling ants or electrical tingling)?

never ☐ hardly noticed ☐ slightly ☐ moderately ☐ strongly ☐ very strongly ☐

Is light touching (clothing, a blanket) in this area painful?

never ☐ hardly noticed ☐ slightly ☐ moderately ☐ strongly ☐ very strongly ☐

Do you have sudden pain attacks in the area of your pain, like electric shocks?

never ☐ hardly noticed ☐ slightly ☐ moderately ☐ strongly ☐ very strongly ☐

Is cold or heat (bath water) in this area occasionally painful?

never ☐ hardly noticed ☐ slightly ☐ moderately ☐ strongly ☐ very strongly ☐

Do you suffer from a sensation of numbness in the areas that you marked?

never ☐ hardly noticed ☐ slightly ☐ moderately ☐ strongly ☐ very strongly ☐

Does slight pressure in this area, e.g., with a finger, trigger pain?

never ☐ hardly noticed ☐ slightly ☐ moderately ☐ strongly ☐ very strongly ☐

(To be filled out by the physician)

never	hardly noticed	slightly	moderately	strongly	very strongly
☐ x 0 = 0	☐ x 1 = ☐	☐ x 2 = ☐☐	☐ x 3 = ☐☐	☐ x 4 = ☐☐	☐ x 5 = ☐☐

Total score ☐☐ **out of 35**

R. Freynhagen, R. Baron, U. Gockel,T.R. Tölle, CurrMed ResOpin Vol 22, 2006, 1911-1920
© 2005 Pfizer Pharma GmbH, Pfizerstr.1, 76139 Karlsruhe, Germany

painDETECT — SCORING OF PAIN QUESTIONNAIRE

Date: ______ **Patient:** Last name: ______ First name: ______

Please transfer the total score from the pain questionnaire:

Total score [ ][ ]

Please add up the following numbers, depending on the marked pain behavior pattern and the pain radiation. Then total up the final score:

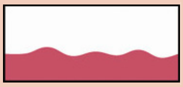	Persistent pain with slight fluctuations	0	
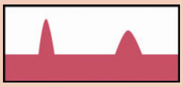	Persistent pain with pain attacks	-1	if marked, or
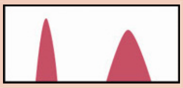	Pain attacks without pain between them	+1	if marked, or
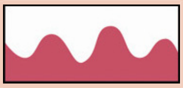	Pain attacks with pain between them	+1	if marked
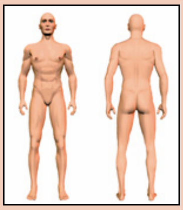	Radiating pains?	+2	if yes

Final score [ ][ ]

Screening Result

on the presence of a neuropathic pain component

negative	unclear	positive
0 1 2 3 4 5 6 7 8 9 10 11 12	13 14 15 16 17 18	19 20 21 22 23 24 25 26 27 28 29 30 31 32 33 34 35 36 37 38
A neuropathic pain component is unlikely (< 15%)	Result is ambiguous, however a neuropathic pain component can be present	A neuropathic pain component is likely (> 90%)

This sheet does not replace medical diagnostics.
It is used for screening the presence of a neuropathic pain component.

DFNS

pain — Pfizer · Analgesic · Innovative · Network

R. Freynhagen, R. Baron, U. Gockel,T.R. Tölle, CurrMed ResOpin Vol 22, 2006, 1911-1920

© 2005 Pfizer Pharma GmbH, Pfizerstr.1, 76139 Karlsruhe, Germany
